# Grapevine Microbiota Reflect Diversity among Compartments and Complex Interactions within and among Root and Shoot Systems

**DOI:** 10.3390/microorganisms9010092

**Published:** 2021-01-02

**Authors:** Joel F. Swift, Megan E. Hall, Zachary N. Harris, Misha T. Kwasniewski, Allison J. Miller

**Affiliations:** 1 Department of Biology, Saint Louis University, 3507 Laclede Ave, St. Louis, MO 63103, USA; zachary.n.harris@slu.edu (Z.N.H.); amiller@danforthcenter.org (A.J.M.); 2 Donald Danforth Plant Science Center, 975 North Warson Road, St. Louis, MO 63132, USA; 3 Division of Plant Sciences, University of Missouri, Agriculture Bldg, 52, Columbia, MO 65201, USA; hallmegan@missouri.edu; 4 College of Agricultural Sciences Department of Food Science, The Pennsylvania State University, 326 Rodney A. Erickson Food Science Building, University Park, PA 16802, USA; mtk5407@psu.edu

**Keywords:** grafting, grapevines, rootstock, sour rot, plant compartments, bacteria, fungi

## Abstract

Grafting connects root and shoot systems of distinct individuals, bringing microbial communities of different genotypes together in a single plant. How do root system and shoot system genotypes influence plant microbiota in grafted grapevines? To address this, we utilized clonal replicates of the grapevine ‘Chambourcin’, growing ungrafted and grafted to three different rootstocks in three irrigation treatments. Our objectives were to (1) characterize the microbiota (bacteria and fungi) of below-ground compartments (roots, adjacent soil) and above-ground compartments (leaves, berries), (2) determine how rootstock genotype, irrigation, and their interaction influences grapevine microbiota in different compartments, and (3) investigate abundance of microorganisms implicated in the late-season grapevine disease sour rot (*Acetobacterales* and *Saccharomycetes*). We found that plant compartment had the largest influence on microbial diversity. Neither rootstock genotype nor irrigation significantly influenced microbial diversity or composition. However, differential abundance of bacterial and fungal taxa varied as a function of rootstock and irrigation treatment; in particular, *Acetobacterales* and *Saccharomycetes* displayed higher relative abundance in berries of grapevines grafted to ‘1103P’ and ‘SO4’ rootstocks and varied across irrigation treatments. This study demonstrates that grapevine compartments retain distinct microbiota and identifies associations between rootstock genotypes, irrigation treatment, and the relative abundance of agriculturally relevant microorganisms in the berries.

## 1. Introduction

Plants have multiple compartments (e.g., roots, leaves, fruits, etc.), each of which offer unique habitats for microorganisms. Plant compartments differ in structural characteristics, micro-environmental conditions, and resource availability which differentially regulate their microbiota [1,2,3,4,5,6]. The microbiota of plant compartments contribute to many essential processes, including nutrient acquisition [6] and adaptation to novel soil conditions [7,8], among others. The composition of microbiota within individual plant compartments reflects dynamic interactions of plant genotype, development, and local environment; however, the extent to which the microbiota in one compartment (e.g., the root) shapes the microbiota of other parts of the plant (e.g., leaves or fruit) is not well known. 

Many factors influence the composition and diversity of plant microbiota including geographic location of the plant, host plant genotype, and biotic and abiotic stresses. Biogeography is a predominant influence on plant microbiota [9,10,11], reflecting unique soil microorganism communities across space [12,13]. Plant host genotype also shapes plant microbiota [14,15,16,17]. For example, within four days of planting aseptically cleaned seeds of nine cotton (*Gossypium hirsutum*) cultivars, Adams and Kloepper [18] found differences in bacterial endophyte composition between cultivars. Genotype-specific microbiota have been identified in numerous other genera including *Helianthus* [19], *Solanum* [20], *Triticum* [16], *Vaccinium* [15], and *Zea* [14,21,22]. The mechanism commonly proposed to explain the observed differences by genotype is altered root exudation profiles [9,23,24]. Plants are also capable of modifying their exudation patterns in response to biotic and abiotic stresses, thereby recruiting microorganisms that can lessen damage to plant health [25,26]. Both drought and pathogen infection cause strong and lasting effects on root exudate profiles that not only impact the individual plant, but also subsequent generations grown in the same location [27,28].

Most studies investigating the factors influencing the microbiome of crops are focused on annual species; however, woody perennials are economically important worldwide [29] and present their own intricacies [30]. For example, the bacterial richness and composition of root-associated microbiota have been shown to shift over the lifetime of perennial plants [31]. Additionally, many woody perennial crops are grafted, a horticultural practice that joins the root system (rootstock) of one individual to the shoot system (scion) of another [32,33]. Grafting physically connects individuals with distinct genomes, allowing genome to genome interactions [34]. Given that plant genotype influences the microbiome, an important question is the extent to which unique genotypes, when grafted together, influence the microbiome of the graft partner.

Grafted grapevines present an ideal model to understand interactions among root and shoot systems and their effects on the microbiota in different plant compartments. The European grapevine (*Vitis vinifera*) is one of the most economically important berry crop species in the world [29]. Since the spread of the root-destroying Phylloxera aphid from North America to Europe in the 1800s [35], grafting of the European species *V. vinifera* and related hybrid scions to phylloxera-resistant rootstocks of North American *Vitis* species and their hybrid derivatives has become ubiquitous in the growing of grapevines. Today more than 80% of vineyards employ grafting [36]. Previous research on grafted grapevines has shown that rootstock genotypes influence scion phenotypes such as leaf ionomic profiles [37,38], shoot gene expression [39], and leaf morphology [40]. Interestingly, grapevine rootstock genotypes have distinct bacterial rhizosphere communities [41,42], and the influence of rootstock genotype on root-associated microbiota appears to increase with vine age [43]. These studies suggest that rootstock genotypes grafted to a common scion retain the ability to recruit distinct root-associated microbiota and this pattern becomes stronger across the lifespan of the grapevine. The extent to which rootstock genotype influences the microbiota of scion compartments (e.g., leaves and berries) is an open question that requires sampling and sequencing from both above- and below-ground compartments of grafted plants. 

Many grapevine rootstocks have been selected primarily for resistance to specific soil-borne pests and pathogens [44]; however, some microorganisms of concern for vine health are associated primarily with the shoot system [45], and the connection between shoot system diseases and root system microbiota remains an open question. For example, sour rot is a late-season bunch rot disease that causes loss of production across many growing regions, particularly those with high rainfall during late stages of berry maturation such as the middle USA [45]. Sour rot is characterized by the oxidation of grape berry skin and loss of berry integrity prior to harvest and is accompanied by a strong odor of acetic acid and the presence of *Drosophila* spp. [46,47]. Berry clusters affected by sour rot result in altered fermentation, changes in wine characteristics, and generally lower wine quality [48,49,50]. This disease has been linked to a four-way interaction between a susceptible grapevine host, *Drosophila* fruit flies, acetic acid bacteria (e.g., *Acetobacter* spp. and *Gluconobacter* spp.) and various yeast species (*Hanseniaspora uvarum*, *Candida* spp., and *Pichia* spp.) [51]. The bacteria and yeast associated with sour rot are commonly found as endo- and epi-phytes of asymptomatic berry clusters [52,53]; however, as symptoms of sour rot develop the microbiota of infected clusters the typically show an elevated abundance of acetic acid bacteria, particularly *Acetobacter* spp. [54]. *Acetobacter* spp. and *Gluconobacter* spp. are able to both oxidize sugars into acetic acid and contribute to higher volatile acidity levels [49,55]. *Hanseniaspora uvarum*, *Candida* spp., and *Pichia* spp. are considered spoilage yeasts in winemaking via production of ethyl acetate and film formation of stored wine [55,56]. Given that the microorganisms implicated in sour rot originate from the grape clusters themselves, we investigated whether particular rootstocks contributed to the enrichment of disease-causing bacterial and yeast populations in the shoot system.

This study aims to advance understanding of the influence of rootstock genotype on microbiota of grafted grapevines. Samples were collected from an experimental vineyard composed of the grapevine cultivar ‘Chambourcin’ as the scion, grown ungrafted and grafted to three different rootstocks. We surveyed multiple replicates of each ‘Chambourcin’ scion/rootstock combination, and collected samples from four distinct compartments for each vine: leaves, berries, root tissue (with rhizosphere attached) and soil. We characterized the bacterial and fungal members of each sample using bacterial and fungal amplicon sequencing. Our objectives were to (1) characterize the microbiota (bacteria and fungi) of below-ground compartments (roots, adjacent soil) and above-ground compartments (leaves, berries), (2) determine how rootstock genotype, irrigation, and their interaction influences grapevine microbiota in different compartments, and (3) investigate abundance of microorganisms implicated in the late-season grapevine disease sour rot (*Acetobacterales* and *Saccharomycetes*).

## 2. Materials and Methods

### 2.1. Experimental Design and Sample Collection

**Figure 1:**
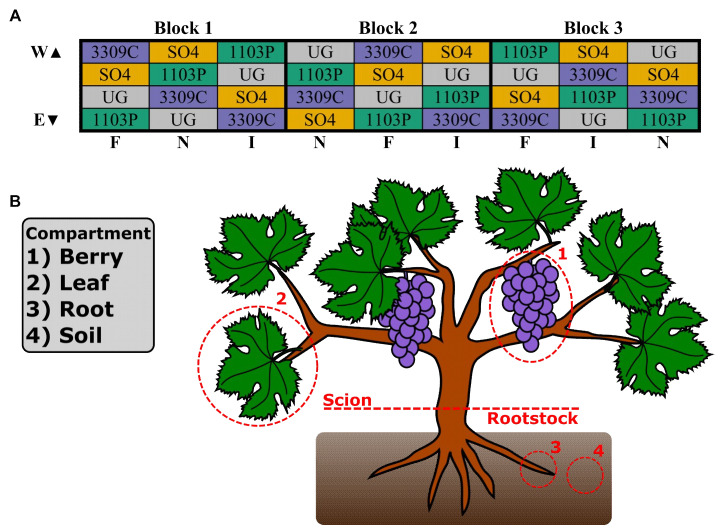
Experimental design. (**A**) Vineyard layout at the University of Missouri Southwest Research Center in Mount Vernon, MO consisting of 288 vines grafted to one of three rootstocks (‘1103P’, 1103 Paulson; ‘3309C’, 3309 Courdec; ‘SO4’, Selection Oppenheim 4) or ungrafted (UG). Each colored cell represents four replicated vines; irrigation treatments (F, Full replacement of evapotranspiration; I, reduced replacement; N, unirrigated) and experimental blocks are listed along the bottom and top of the grid, respectively. (**B**) Depiction of a grafted grapevine with bulk soil and different compartments highlighted; numbers correspond to legend (left).

The rootstock experimental vineyard used in this study was established in 2009 at the University of Missouri Southwest Research Center Agricultural Experimental Station in Mount Vernon, Missouri USA (37.074 N, 93.879 W). The ~8000 m^2^ vineyard is planted with vine rows running from east to west, and experiences mean annual rainfall of 1066.8 mm, mean annual temperature of 15.6 °C [57], and mean annual growing degree days of 461 (URL: https://gwi.missouri.edu/IPMreports/2019-no8_GrowingDegree.htm). The soil is a combination of sandy loam, silt loam, and loam with an average pH of 7 (supplementary Table S1) [57]. A subplot within the vineyard consists of the *Vitis* interspecific hybrid, ‘Chambourcin’, growing ungrafted and grafted to three different rootstocks: 1103 Paulson (‘1103P’; *Vitis berlandieri* × *V. rupestris*), 3309 Courdec (‘3309C’; *V. riparia* × *V. rupestris*), and Selection Oppenheim 4 (‘SO4’; *V. berlandieri* × *V. riparia*). Each scion/rootstock combination is replicated 72 times in a randomized block experimental design (Figure 1). Two additional vines are planted at the beginning, end, and middle point of a row to buffer experimental vines against edge effects and irrigation risers. One of three different irrigation treatments are applied by row in a randomized order (1) no irrigation, (2) full replacement of evapotranspiration as calculated on a rolling weekly basis, and (3) half replacement of evapotranspiration (Figure 1). This vineyard undergoes chemical spray applications for fungal diseases (e.g., downy mildew, black rot, Botrytis bunch rot, and Phomopsis cane and leaf spot), consistent with industry practices. Without fungicide applications, vines become infected with fungal pathogens, resulting in the loss of fruit, leaves, and the potential death of the vines. 

Samples were collected from 72 vines (four scion/rootstock combinations × three irrigation treatments × six replicates per scion/rootstock/irrigation combination) on September 18, 2018, coinciding with the timing of commercial harvest, E-L number 38 [58]. For each vine, samples were collected from bulk soil, rhizosphere, leaves, and one berry cluster (Figure 1). Bulk soil was collected from at least 3 cm below ground level with a shovel or hand trowel at the base of the stem, and passed through a sieve (American Standard No. 16; 1.18 mm pore size). Roots were collected at a depth of 20-30 cm by manually removing roots from the soil. Five leaves 8–12 cm in diameter were collected at roughly the same position along the shoot and height on the vine. Berries were collected as an intact cluster, free of damage or apparent disease symptoms. All collections were made using sterilized equipment and conditions. For each treatment (Figure 1; colored cells), samples were collected from the two inner vines of a set of four plants. In block three, samples were collected on an individual vine basis, whereas in blocks one and two samples were homogenized per treatment (rootstock genotype) in the field. In total, 192 samples (48 from each compartment type) were collected in polyethylene bags, placed in a cooler with dry ice for transport, and stored at −80 °C until DNA extraction.

### 2.2. DNA Extraction, Amplification, and Sequencing

DNA extractions were performed at the Danforth Plant Science Center (Saint Louis, MO, USA) using the DNeasy PowerSoil Kit (Qiagen, Germantown, MD, USA) following the manufacturer’s protocol with two modifications; plant tissue samples had 150 mg per extraction and a 10-min (70 °C) incubation step prior to homogenization with a bead mill (Retsch MM 400). The surfaces of the samples were not sterilized and thus contained both endo- and epi-phytes. For berry cluster samples, two berries of equal size were placed into a sterilized stainless-steel grinding jar with a stainless-steel ball (Retsch, Haan, Germany), frozen in liquid nitrogen, and then pulverized prior to homogenization. Leaf and root samples were finely chopped with a sterile scalpel prior to homogenization. All extracts were quantified using a DS-11 spectrophotometer (Denovix, Wilmington, DE, USA).

Library preparation and amplicon sequencing was conducted at the Environmental Sample Preparation and Sequencing Facility at Argonne National Laboratory (Lemont, IL USA). The V4 region of the 16S rRNA gene, 515F-806R [59,60] and the internal transcribed region, ITS1f-ITS2 [61] were separately amplified with region-specific primers that include sequencer adapter sequences used in the Illumina flowcell [62,63]. The forward amplification primer contained a twelve base barcode sequence to allow pooling of samples onto a single sequencing lane [62,63]. To each 96-well plate two negative controls (extraction and PCR) and one positive control, ZymoBIOMICS™ Microbial community DNA standard (Zymo Research, Irvine, CA, USA) were added. Each 25 µL PCR reaction contained 9.5 µL of MO BIO PCR Water (Qiagen), 12.5 µL of AccuStart II PCR ToughMix (2× concentration, 1× final, QuantaBio, Beverly, MA, USA), 1 µL barcode tagged Forward Primer (5 µM concentration, 200 pM final), 1 µL Reverse Primer (5 µM concentration, 200 pM final), and 1 µL of template DNA. For 16S rRNA gene PCRs peptide-nucleic-acid blockers (PNA Bio, Thousand Oaks, CA, USA) were used to reduce non-target amplicons originating from chloroplast and mitochondria [64], 1 µL (5 µM concentration, 200 pM final) of each was used. The conditions for the 16S rRNA gene PCR were: 94 °C for 3 min, with 35 cycles at 94 °C for 45s, 50 °C for 60 s, and 72 °C for 90 s; with a final extension of 10 min at 72 °C. The conditions for the ITS PCR were: 94 °C for 1 min, with 35 cycles at 94 °C for 30 s, 52 °C for 30 s, and 68 °C for 30 s; with a final extension of 10 min at 68 °C. Amplicons were then quantified using PicoGreen (Thermo Fisher Scientific, Waltham, MA, USA) and a plate reader (Infinite 200 PRO, Tecan, Männedorf, CHE). Once quantified, volumes of each of the products were pooled in equimolar amounts. This pool was then cleaned using AMPure XP Beads (Beckman Coulter, Brea, CA, USA), and quantified using a fluorometer (Qubit, Thermo Fisher Scientific). After quantification, the molarity of the pool was determined and diluted down to 2 nM, denatured, and then diluted to a final concentration of 6.75 pM with a 10% PhiX spike. Sequencing was conducted on a MiSeq (Illumina, San Diego, CA, USA), 2 × 151 bp PE for 16S rRNA gene and 2 × 250 b PE for ITS using customized sequencing primers and procedures [63].

### 2.3. Bioinformatic Processing

Data processing and preliminary analyses were conducted in QIIME2 v.2019.1 [65]. Samples were demultiplexed (*qiime demux emp-paired*) according to barcode sequence. For 16S rRNA gene sequences, the QIIME2 plugin DADA2 [66] was used to denoise, dereplicate, and filter chimeric sequences. The first 13 nucleotides (nt) of each sequence (forward and reverse) were trimmed and truncated at 150 nt to remove lower quality bases. For ITS sequences, Cutadapt [67] was used to remove primer sequences prior to using the DADA2 plugin. The first 12 nt of the 3′ end of each sequence were trimmed, the 5′ end of sequences were not truncated to preserve biologically relevant length variation [68], and a max expected error rate of 3 was used. The result of DADA2 processing was a table of Amplicon Sequence Variants (ASV). Workflows using ASVs perform similar to operational taxonomic unit (OTU) based methods with the benefits of comparability across studies (using the same primers) and increased taxonomic resolution [69,70]. Taxonomic classification of ASVs was conducted with a naive Bayes classifier trained on either the SILVA v.132 [71], trimmed to the V4 region, or UNITE database v.8.0 [72] for bacteria and fungi, respectively. Bacterial and fungal ASVs not assigned to a phylum were removed along with bacterial ASVs assigned to mitochondria or chloroplasts. ASVs with less than 0.1% of the total filtered reads, which tend to inflate diversity metrics [73], were removed. Negative and positive control samples were processed in a similar manner, but we did not remove bacterial ASVs assigned to mitochondria or chloroplasts or filter those at less than 0.1% of filtered reads (Figure S1).

### 2.4. Statistical Analyses

Samples were rarefied to 1500 and 5000 sequences for bacteria and fungi, respectively. Alpha diversity and beta diversity metrics were calculated in phyloseq v.1.26.1 [74]. For bacterial samples, we calculated three alpha diversity metrics (inverse Simpson’s, Shannon’s, and Faith’s phylogenetic diversity indices) and the beta diversity metric UniFrac (both weighted, which accounts for relative abundance, and unweighted), which considers phylogenetic relatedness between samples in calculations [75]. For fungal samples, we calculated inverse Simpson’s and Shannon’s diversity indices and Bray–Curtis dissimilarity, a beta-diversity metric. Principal coordinates analysis was used to visualize sample relationships. Venn diagrams were generated by determining the intersection of ASV lists per compartment (URL: http://bioinformatics.psb.ugent.be/webtools/Venn/).

Statistics were calculated in base R v.3.6.1 [76] and in various R packages. Figures were generated with ggplot2 v.3.2.1 [77] and arranged using ggpubr (URL: https://github.com/kassambara/ggpubr/). In order to test for significant differences between alpha diversity means by compartment, a Tukey’s honestly significant difference (HSD) test was applied. Linear models and PERMANOVA tests were run using the formula: response variable ~ Rootstock genotype × Compartment × Irrigation + Block. For each alpha diversity metric, a linear model was fit using lm and assessed using type-III ANOVA with the car package v.3.0-3 [78]. Beta diversity metrics were subjected to PERMANOVA tests with adonis in the vegan package v.2.5-6 [79,80] using 10,000 permutations. 

In order to investigate the distribution of bacterial and fungal taxa associated with the late-season bunch rot disease sour rot, we used the *subset_taxa* function in phyloseq to extract ASVs from the rarified datasets that were assigned to the bacterial family *Acetobacterales* or the fungal class *Saccharomycetes*. Relative abundance of these taxa across compartments, and for rootstock and irrigation treatments, were visualized using boxplots. We ran linear models using the full experimental design formula (Rootstock genotype × Compartment × Irrigation + Block) with the abundance of *Acetobacterales* or *Saccharomycetes* as the response variable. We conducted post-hoc comparisons of means using the emmeans package v.1.4.5 [81], correcting for multiple comparisons, to understand which comparisons of significant factors were driving the abundance of these taxa. To test for correlation between the relative abundance of *Acetobacterales* and *Saccharomycetes*, we conducted a Spearman rank-based correlation using the *stat_cor* function from the ggpubr package.

In order to determine whether each rootstock had a predictable impact on the microbiota we used a two-pronged approach: (1) random forest to identify which factors were classifiable; and (2) differential abundance analysis to identify individual microorganisms. First, for machine learning we used ranger’s implementation of random forest v.0.11.2 [82] and tuned hyperparameters (number of trees, minimum node size, and number of features available at each node) with Caret v.6.0-84 [83] on 80% of the dataset (20% withheld for testing). The optimal hyperparameters were selected by iteratively assessing the performance on out-of-bag samples for a given parameter set. We then used this final model to predict the label, either rootstock or compartment or both, on the withheld testing data assessing the prediction accuracy. Data were visualized using tile plots from the output confusion matrix. Second, for differential abundance analysis, we used unrarefied reads and removed ASVs that were not represented by a depth of least 25 reads in more than 10% of the samples to conduct differential abundance modeling with DESeq2 v.1.24.0 [84]. DESeq2 was fit using the following model: response variable ~ Rootstock genotype (R) + Compartment (C) + Irrigation (I) + R × C + I × R + I × C + Block (B). For each factor we extracted the number of ASVs that showed a significant pattern of differential abundance as well as their fold changes (Log_2_) to generate summary plots. 

## 3. Results

We generated bacterial (16s rRNA gene) and fungal (ITS) amplicon sequence data for bulk soil, roots, leaves and berries of 72 grapevines. For bacteria, this study produced 13,003,903 reads, of which 10,647,004 reads remained following quality and chimera filtering. Reads collapsed into 20,602 bacterial ASVs. In order to remove host contamination, bacterial ASVs assigned to mitochondria and chloroplasts were removed (9.1% and 31.4% of reads, respectively). ASVs with less than 0.1% mean sample depth were removed, resulting in 8199 ASVs and 6,155,632 reads for bacteria. We used rarefaction to normalize the number of reads per sample, 1500 reads per sample, resulting in the removal of seven samples (Supplementary Figure S2A). For fungi, we generated 11,434,967 reads; 5,748,318 reads passed quality and chimera filtering, and these collapsed into 2475 ASVs. After mean sample depth filtering the resulting fungal dataset included 1429 ASVs comprised of 5,735,409 reads. Finally, we rarefied fungal samples at 5000 reads, resulting in 11 samples being removed (Supplementary Figure S2B).

### 3.1. Bacterial and Fungal Community Composition and Richness Strongly Associate with Plant Compartment

**Figure 2:**
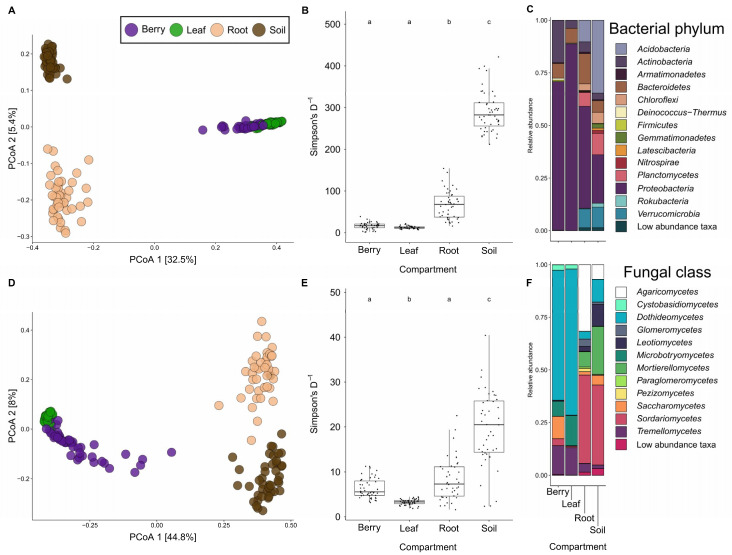
Principal coordinates analysis illustrates strong clustering of samples by compartment for (**A**) bacterial (unweighted UniFrac: PERMANOVA *F*_3176_ = 40.7, *p* < 0.001) and (**D**) fungal (Bray–Curtis: PERMANOVA *F*_3176_ = 74.3, *p* < 0.001). Inverse Simpson’s Diversity index of microorganism compartments for (**B**) bacteria and (**E**) fungal samples show a relative increase in diversity from above-ground to below-ground compartments. Taxonomic barplots for (**C**) bacterial phyla and (**F**) fungal classes reveals differences in microorganism community composition and structure by compartment.

Principal coordinates analyses (PCoA) for bacterial unweighted UniFrac distances and fungal Bray–Curtis dissimilarity show clear clusters by compartment, with the first axis separating above and below-ground compartments, the second axis separating soil and roots, and the third axis begins to pull apart berries and leaves (Figure 2A,D; For axis three see Supplementary Figure S5). PERMANOVA tests corroborated the PCoA results: for bacteria, compartment was the only significant factor explaining 40.7% of the variance (unweighted UniFrac: *F*_3176_ = 40.7, *p* < 0.001; Table 1). Fungal communities showed significant effects of compartment (explaining 55.0% of variance), rootstock (1.6% of variance) and rootstock x compartment interaction (3.2% of variance; Table 1).

Below-ground compartments, soil and roots, were more diverse than above-ground compartments, leaves and berries, for both bacteria and fungi (Figure 2B,E and Supplementary Figure S3). Bacterial communities were most diverse in soil, then roots, followed by berries and leaves, with mean inverse Simpson’s diversity values of 634.1, 437.9, 56.6, and 52.8, respectively (Figure 2B). Fungal communities showed similar patterns; however, fungal communities in berries were significantly more diverse than fungal communities in leaves with mean inverse Simpson’s diversity values 110.1, 66.0, 47.9, 29.1 for soil, roots, berries and leaves, respectively (Figure 2E,D). For bacteria and fungi, linear models run on alpha diversity metrics demonstrated that compartment was significant across all metrics (*p* < 0.001; Supplementary Tables S2 and S3). Venn diagrams showed a similar pattern to the alpha diversity metrics, while the intersections revealed that many ASVs are shared in multiple tissues and the soil for bacteria and fungi (Supplementary Figure S4). Interestingly, while soil contained the highest number of ASVs, a sizable number of ASVs were found only in association with plant tissues and not in soil (~26% and ~32% for bacteria and fungi, respectively).

Differences in taxonomic profiles were apparent between compartments (Figure 2C, F). Bacterial samples of leaves and berries were dominated by Proteobacteria, with smaller proportions of Actinobacteria and Bacteroidetes (Figure 2C). Root and soil compartments were considerably more diverse but still showed a large proportion of the phylum Proteobacteria; additional phyla recovered in root and soil compartments included Acidobacteria (34.6% soil vs. 10.3% root), Bacteroidetes, Verrucomicrobia, Planctomycetes, Actinobacteria, and Chloroflexi. Fungal samples of leaves and berries were also dominated by a few taxonomic classes, Dothideomycetes, Agaricomycetes, and Mortierellomycetes (Figure 2F). Berries showed the largest abundance of Saccharomycetes in comparison to other compartments (10.6% berries vs. 4.5% soil). Root and soil compartments showed several additional fungal classes including Sordariomycetes, Tremellomycetes, Microbotrymycetes, Leotiomycetes, Cystobasidiomycetes, Glomeromycetes, Pezizomycetes, and Paraglomeromycetes. 

### 3.2. Rootstock Genotype and Irrigation have Subtle Effects on Community Patterns 

**Figure 3:**
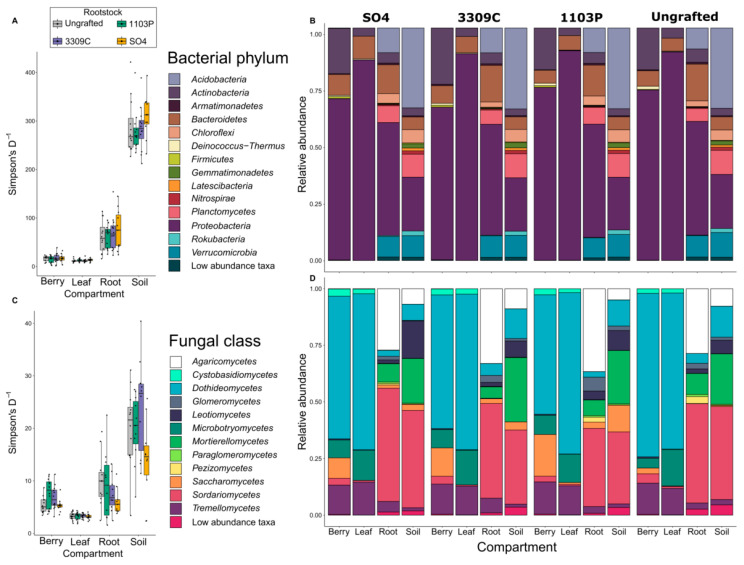
Inverse Simpson’s Diversity index and taxonomic barplots for microorganism compartments across rootstock genotypes for (**A**,**B**) bacteria and (**C**,**D**) fungal samples illustrate that rootstock has only subtle effects on patterns of diversity and composition of microorganism communities of grapevines.

Bacterial and fungal diversity, including alpha and beta diversity measured within compartments and across compartments within vines of specific scion/rootstock combinations, were generally similar regardless of rootstock (Figure 3). Alpha diversity metrics for bacteria showed no significant effect of rootstock genotype (Supplementary Table S2). Beta diversity analysis showed similar patterns to alpha diversity metrics (Table 1); for bacteria, rootstock and its interactions were non-significant. For fungi, inverse Simpson’s diversity index differed by rootstock (Supplementary Table S3); post-hoc testing revealed that this variation was significantly described by rootstock when comparing ‘3309C’- and ‘SO4’-grafted vines in the soil samples (‘3309C’ vs. ‘SO4’ soil: 10.369, t_127_ = 4.817, *p* < 0.0001). Fungi showed significant effects of rootstock and its interaction with compartment; (Bray–Curtis: rootstock *F*_3173_ = 2.2, rootstock×compartment *F*_9167_ = 1.5; Table 1). Although irrigation treatments were imposed on the vines in this study (N; None, F; Full, and I; half replacement of evapotranspiration; Figure 1A), these did not have a large impact on the microorganism communities. Linear models fit to alpha and beta diversity metrics for both bacteria and fungi did not find irrigation to be a significant predictor (Table 1, Supplementary Tables S2 and S3).

Taxonomic profiles were similar across rootstock genotypes for each compartment (Figure 3B,D). For bacteria, at the phylum level, the phyla identified and relative abundance of each was largely controlled by the compartment as opposed to the rootstock genotype (Figure 3B). For fungi, the pattern was mostly the same, with the greatest differences in taxa and relative abundance of classes being related to compartment (Figure 3D). *Saccharomycetes* showed patterning by rootstock, with all grafted plants showing an increased abundance (Figure 3D). After post-hoc testing we found that berries from the rootstock ‘1103P’ showed significantly higher abundance than ungrafted vines and grafted ‘SO4’ vines (‘1103P’ vs. Ungrafted berries: *p* < 0.001 and ‘1103P’ vs. ‘SO4’: *p* = 0.043).

### 3.3. Rootstock and Irrigation Influence Microbiota of Winemaking Relevance 

While characterizing the microbiota of different rootstocks, we observed that samples containing a high relative abundance of *Saccharomycetes* also generally had increased relative abundance of the bacterial order *Acetobacterales*. These taxa, while ubiquitous in and on grape berries, are also causal organisms of the late-season bunch rot disease sour rot and are relevant to winemaking. In order to more thoroughly investigate the impact of rootstock on these agronomically and viticulturally important taxa, we extracted members of the bacterial order *Acetobacterales* and the fungal class *Saccharomycetes* from the rarified dataset. The extracted *Acetobacterales* including 30 species representing seven genera, with most sequence reads comprising members of *Acetobacter* and *Gluconobacter* (6.1% and 83.4%). The extracted *Saccharomycetes* represented 48 species from 12 genera, with most concentrated in the genera *Hanseniaspora* and *Pichia* (32.9% and 25.7%). 

**Figure 4:**
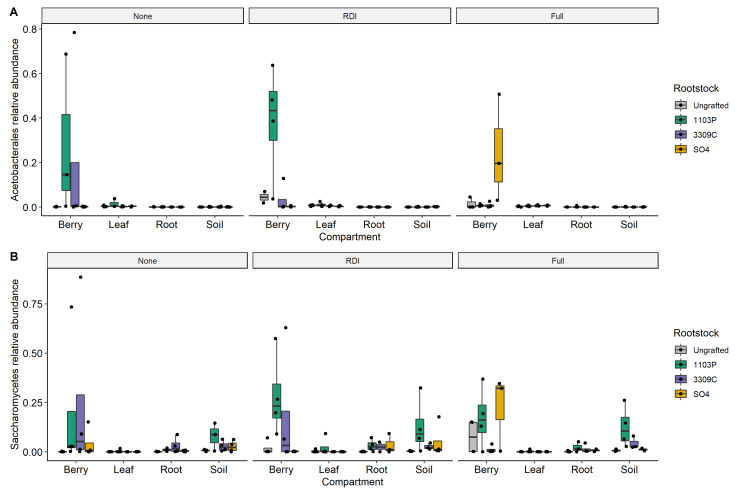
The abundance of (**A**) *Acetobacterales* and (**B**) *Saccharomycetes* are influenced by complex interactions of rootstock genotype, irrigation treatment, and plant compartment. Panels delineate irrigation treatments (None, unirrigated; RDI, reduced replacement of evapotranspiration; Full, Full replacement) and rootstock genotypes correspond to colors in the legend.

Linear models demonstrated that rootstock and its interactions significantly influence the relative abundance of *Acetobacterales* and *Saccharomycetes* (Table 2). *Acetobacterales* relative abundance was significantly influenced by the three-way interaction of rootstock, compartment, and irrigation (R × C × I; *p* = 0.002; Table 2). Post-hoc testing showed that all variation was attributable to the berry compartment, with other compartments showing no significant comparisons (Figure 4A). Within the full irrigation treatment, berries of ‘SO4’ grafted vines had elevated relative abundance of *Acetobacterales* in comparison to other vines (SO4 vs. Ungrafted *P* = 0.009, ‘SO4’ vs. ‘1103P’ *p* = 0.003, and ‘SO4’ vs. ‘3309C’ *p* = 0.004). Whereas berries of ‘1103P’ grafted vines had elevated relative abundance under reduced (‘1103P’ vs. Ungrafted *P* < 0.001, ‘1103P’ vs. ‘3309C’ *p* < 0.001, ‘1103P’ vs. ‘SO4’ *p* < 0.001) and no supplemental irrigation treatments (‘1103P’ vs. Ungrafted *P* = 0.001, ‘1103P’ vs. ‘SO4’ *p* < 0.001). *Saccharomycetes* relative abundance was influenced by the interaction of rootstock and irrigation (R × I; *p* = 0.007; Table 2). Post-hoc testing showed only a single significant comparison for the interaction, ‘1103P’ grafted vines had elevated *Saccharomycetes* relative abundance compared to ungrafted vines within the reduced irrigation treatment (‘1103P’ vs. Ungrafted *P* = 0.048; Figure 4B); however, ‘1103P’ vines compared to ungrafted vines across irrigation treatments showed much higher significance (‘1103P’ vs. Ungrafted *P* = 0.006). The relative abundance of *Acetobacterales and Saccharomycetes* for berries were strongly positively correlated (Spearman correlation = 0.735, *p* < 0.001; Figure S6). 

### 3.4. Machine Learning and Differential Abundance Analyses

**Figure 5:**
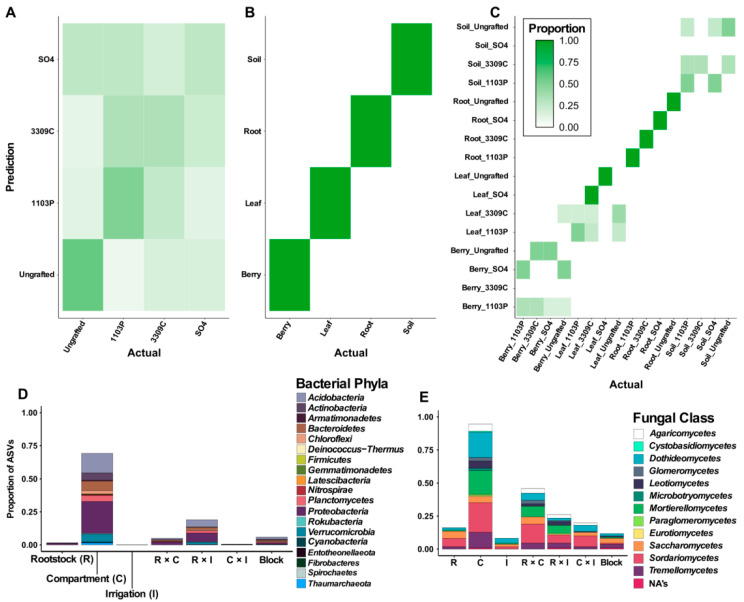
Machine learning was able to distinguish between (**A**,**B**) the compartment from which the sample was collected but not rootstock genotype to which the vine was grafted and (**C**) when compartment and rootstock were jointly predicted only compartment was able to be predicted accurately. DESeq2 analysis found that multiple sources of variation were responsible for the patterns of differential abundance for amplicon sequence variants (ASVs) of (**D**) bacteria phyla and (**E**) Fungi classes.

For machine learning-based classification we tested the random forest algorithm on both regions (16S rRNA gene and ITS) both separately and together. We found that the accuracy was similar for each region and the combined dataset; consequently, here we report the results of the combined dataset. After hyperparameter tuning of the random forest models (Supplementary Figure S7 and Table S4) performance on the combined 16s rRNA gene and ITS testing data showed high accuracy in classifying samples to compartment but not to rootstock (98% and 35% accuracy, respectively; Figure 5A–C; Supplementary Tables S5 and S6). When compartment and rootstock were jointly classified, we found that accuracy was higher (50% accuracy) with most samples being correctly assigned to compartment, but rootstock genotypes assignments were stochastic (Supplementary Table S7). Similarly, we were unable to accurately classify which irrigation treatment was applied to a given sample (45% accuracy; Supplementary Figure S8). These data corroborate other analyses that point to compartment as the primer determinant of microbiota diversity within a vine. 

Differential abundance analysis in DESeq2 illustrated that ASVs are associated with multiple factors in the experimental design (Figure 5D,E). After filtering ASVs not represented at a depth of least 25 reads in more than 10% of the samples, we were left with 757 bacterial and 111 fungal ASVs. Overall a greater proportion of fungal taxa respond to each of the factors more than bacteria. We found that bacteria and fungi generally followed the same rank order in regards to the proportion of differentially abundant ASVs responding to each source of variation (Figure 5D,E). Compartment showed the largest proportion of differentially abundant ASVs with 69.4% of bacterial and 94.6% of fungal ASVs. For bacteria, interactions between sources of variation showed larger proportions of differentially abundant ASVs than the other main effects (R = 1.5%, I = <1%, R × C = 5.0% and R × I = 19.0%, C × I = <1%; Figure 5D). The main bacterial phyla that showed differential abundance with respect to the experimental design were Proteobacteria, Acidobacteria, Bacteroidetes, Planctomycetes, Actinobacteria, and Chloroflexi (Figure 5D). Fungi also showed a greater proportion of differentially abundant ASVs associated to interaction effects (R = 16.2%, I = 8.1%, R × C = 45.9%, R × I = 19.8%, C × I = 26.1%; Figure 5E). The main fungal classes that responded to the different parts of the experimental design were Dothideomytcetes, Sordariomycetes, Mortierellomycetes, Tremellomycetes, Agaricomycetes, Saccharomycetes, and Leotiomycetes (Figure 5E). 

The extracted absolute Log_2_ fold change value of each ASV significantly responding to the experimental design was used to compare the effect sizes of each source of variation. The mean Log_2_ fold change for each of the sources of variation were similar, except for block which had a lower Log_2_ fold change value 1.20 ± 0.20 for bacteria and 1.33 ± 0.28 for fungi (Supplementary Figure S9). Post-hoc tests showed that for bacteria all comparisons to block were significant. For fungi all comparisons to block were again significant, but we also observed that compartment had a significantly larger Log_2_ fold change mean than rootstock and irrigation (+1.54 and +1.62, respectively; Supplementary Figure S9).

## 4. Discussion

The grapevine microbiome consists of multiple compartments (soil, root/rhizosphere, leaves, and berries) each of which host unique communities of bacterial and fungal taxa. Within the study, we found that alpha and beta diversity indices for both bacteria and fungi, varied primarily by grapevine compartments (Figure 2). Although rootstock genotype exhibits subtle impacts on patterns of bacterial and fungal diversity (Figure 3), additional microorganisms were uncovered that showed differential abundance across the factors and interactions within the experimental design (Figure 5). Notably, in the berry compartment, patterns of relative abundance in the viticulturally important *Acetobacterales* and *Saccharomycetes* bear a signature of rootstock and irrigation (Figure 4). 

We found that compartments were distinct from one another with the abundance of particular bacterial phyla and fungal classes changing dramatically between compartments (Figure 2). In accordance with Zarraonaindia et al. [85], bacterial community structure changed most dramatically between above- and below-ground samples (Figure 2B). Fungal communities also experienced a strong change between above- and below-ground samples (Figure 2D) consistent with prior studies [86]. The large differences observed between compartments allowed for abundance-based machine learning to easily predict the compartment of a sample (Figure 5B). As we show, the compartments each possess distinct microbiota that might be impacted in diverse ways by the rootstock genotypes.

Soil serves as the main reservoir of microorganisms within agricultural fields [1,6,87,88,89], as such we expected each of the plant compartments would contain a fraction of the diversity found within the soil. In this study we found that a majority of both the bacterial and fungal ASVs were found in or in association with soil, 74% and 68% respectively (Supplementary Figure S4). The ASVs not associated with soil could have originated from the atmosphere via rainfall or wind [89], from the microbiota present in the cutting used in the establishment of the vineyard, or could be exceedingly rare in the soil so as to avoid detection. Atmospheric microorganism originate from multiple sources, including the local vegetation surfaces (i.e., plant phyllosphere), soils, and bodies of water [90,91,92,93]. Local atmospheric conditions (e.g., relative humidity, temperature, and etc.) and seasonal variability have been found to influence atmospheric microbial community composition [91,94,95,96], but strong effects are also associated with the type of land-use [90]. Hyma and Fay [97] showed that *Saccharomyces cerevisiae* ecotypes were readily dispersed from oak trees to grapevines in surrounding vineyards. Thus, it is possible some of the microorganisms we identified, only in association with the plant, originated from the surrounding environment and might be important to the formation of above-ground microorganism communities.

The difference in microbial diversity observed between below and above-ground compartments can be attributed to multiple factors such as above-ground compartment structure and chemistry, fluctuating water availability, harsh climatic conditions, and limited nutrient access, all of which influence the micro-environments of the above-ground compartments [5]. For bacteria and fungi inhabiting above-ground compartments, water availability is paramount to survival and fluctuates greatly with environmental conditions [98,99]. For instance, the level of relative humidity was found to positively correlate with fungal abundance and richness in the air and on leaf surfaces [100]. Harsh climatic conditions, such as exposure to UV radiation [101], further limit the abundance and richness of microorganisms on the surface of above-ground tissues. In addition, nutrients on the surface of above-ground tissues are scarce and more heterogeneously distributed as compared to nutrients available to soil- and rhizosphere dwelling microorganisms [102]. These factors have been shown in other studies to limit the bacterial and fungal diversity and likely contribute to the observed diversity levels in both leaf and berry compartment samples in this study.

Our study is the first that attempts to simultaneously assess the root and shoot systems of grapevines with an eye toward understanding the influence of grafting on the shoot system microbiota. Previous research has shown that rootstock genotypes influence the community that associates with the rhizosphere and root endosphere of the grapevine [41,42,43]. Furthermore, cultivar-specific differences in the microbial communities of berries and leaves have been recorded [11,103,104,105,106]. Our results show that grafting and the different rootstock genotypes had a subtle effect on the diversity indices of the different compartments of the vine. Alpha (Figure 3A,C; Supplementary Tables S2 and S3) and beta diversity indices (Table 1) indicate a non-significant influence of rootstock and its interactions for bacterial communities. However, for fungal communities a small but significant percentage of variance was explained by rootstock and rootstock by compartment interaction (1.6% for R and 3.2% for R × C fungal Bray–Curtis; Table 1). This indicates that there is a core microbiome of the grapevines within this vineyard, which is variable by compartment, but is largely conserved across different rootstocks and the process of grafting. However, there are signatures of rootstock and rootstock by compartment interaction in fungal communities. This could be the result of multiple factors. First, within our study we did not separate the rhizosphere and root endosphere compartments, opting to grind the root tissue with the rhizosphere still adhered. This could obscure the detection of an effect of rootstock genotype if these compartments, rhizosphere and root endosphere, respond differently. Second, the scion we sampled from, ‘Chambourcin’, is of complex hybrid origin, whereas the previous studies made use of *Vitis vinifera* cultivars [‘Barbera’, ‘Tempranillo’, and ‘Lambrusco’; 41–43]. Currently, more work is required to understand how the scion portion of a grafted plant influences the microbiota of the rootstock and other compartments of the vine.

While irrigation treatments were applied to the vines throughout the growing season, we did not find a large impact of irrigation on patterns of microbial diversity. There are two different scenarios that can explain these results. The first possibility is that the microbiota associated with the different tissues of the grapevine are unimpacted by the amount of water that the grapevine receives. This is unlikely as previous research has shown that drought typically alters the microbiome across many plant species [17,25]. For example, in a study on Sorghum, under drought conditions rhizosphere and root microbiota showed enrichment of monoderms, bacteria with a single membrane and thick peptidoglycan cell wall, which likely provides increased desiccation resistance [107]. The second possibility is that the amount of seasonal precipitation received during the growing season was enough to obscure some of the signal from the irrigation treatment. Previous research in this experimental vineyard from prior years, have shown that irrigation treatments have impacted multiple phenotypes, including, physiologic measurements in 2014–2015 [57] and the ionome and morphology of leaves in 2014–2016 [40]. However, the effects of irrigation in the past in this vineyard were weaker than other parts of the experimental design (e.g., development and rootstock genotype) and these studies collected measurements or samples across a wider portion of the growing season than the current study. In the month prior to the sampling date (18 August–18 September 2018) the vineyard experienced an average precipitation of 0.56 ± 1.27 cm of precipitation per day with three days that experienced greater than 3 cm of precipitation (Missouri Historical Agricultural Weather Database URL: http://agebb.missouri.edu/weather/history/). This amount of precipitation exceeded the amount of water the soil was expected to lose from evapotranspiration, given the environmental conditions, leading to vines not experiencing severe water stress.

We found both *Acetobacterales* and *Saccharomycetes* were influenced by complex interactions involving multiple factors including rootstock, irrigation, and compartment (Figure 4). For *Acetobacterales*, we found a significant three-way interaction between rootstock, irrigation, and compartment (Table 2). This was driven by an elevated relative abundance in berries for vines grafted on the rootstock ‘1103P’ in the unirrigated and reduced irrigation treatments and by ‘SO4’-grafted vines in the full irrigation treatment (Figure 4A). For *Saccharomycetes*, we found a significant interaction between rootstock and irrigation but post-hoc testing showed only a single significant comparison whereas the main effect of rootstock showed more significant post-hoc comparisons and explained a similar amount of the variance (7.69% and 7.07%, respectively; Table 2). *Saccharomycetes* across irrigation treatments showed elevated relative abundances in grafted vines compared to ungrafted (Figure 4B). We also observed a strong positive correlation between the relative abundance of *Acetobacterales* and *Saccharomycetes* for the berry compartment (Supplementary Figure S6). 

The correlation between *Acetobacterales* and *Saccharomycetes* abundance in the berry indicates that these groups may interact [51,54]. Both of the microorganism groups tended to be in higher abundance in the berry compartment of grafted grapevines. This indicates that either grafting or the rootstock genotypes in this study have the ability to influence the abundance of the microorganisms implicated in sour rot. Previous work has shown that plant genotypes possess unique root exudate profiles [23,108,109] and that these exudates shape microbial communities [24,110]. Thus, metabolomic analysis would be useful to corroborate the association that we observed with ‘1103P’ and the grafted vines in comparison to ungrafted vines. It is possible that rootstocks produce compounds, whether specific to genotype or as an effect of grafting, that contribute to elevated abundance of *Acetobacterales* and *Saccharomycetes* either directly through specialized substrates or indirectly via suppression of competition with other microorganisms. With the recent emergence of insecticide resistance in populations of *Drosophila* reported in the Finger Lakes region, New York [111], additional understanding of the dynamics of the constituents of sour rot to common vinicultural techniques are a critical need. 

*Acetobacterales* and *Saccharomycetes* also contribute to wine quality. The acetic acid bacteria we recovered were mostly of the genera *Gluconobacter* and *Acetobacter*. These are aerobic bacteria able to oxidize sugars to acetic acid when berries are damaged [51] and during wine production when products are exposed to oxygen [49,112,113]. Non-*Saccharomyces* yeasts have been investigated for the properties in winemaking [114,115,116,117]. The non-*Saccharomyces* yeast we recovered were mostly *Hanseniaspora* and *Pichia,* both of which are generally considered spoilage yeasts when they are dominate within a fermentation [55] but can be beneficial if allowed to perform initial fermentation and then followed up with an inoculation of a strong fermenting yeast species (e.g., *Saccharomycetes cerevisiae*; 121). *Hanseniaspora* in fermentations was found to contribute to the accumulation of acetic acid and ethyl acetate [115,116,118]. Similarly, *Pichia* can contribute to higher levels of ethyl acetate in wines [118] although a study using simple culture mediums show conflicting results [119]. These genera have also been used to successfully enhance wine fermentations when co-inoculated with another fermenting yeast, due to their β–glucosidase activity allowing for higher productions of volatiles [120,121,122]. Thus, our results illustrate that the treatments imposed on the vines in the vineyard, namely grafting and irrigation, can lead to microbial changes in the berries which could have further implications on the fermentation microbiome.

## 5. Conclusions

Grafting, the process by which plant parts are fused together, provides a unique avenue to explore the ways in which root and shoot systems interact. In grapevines, we have shown that plant compartments retain unique bacterial and fungal communities regardless of the whether they were grafted or to which rootstock genotype they are grafted. Indicating that the environmental conditions that microorganisms are exposed to within different parts of the plant are paramount. We found that rootstock genotype, irrigation, and their interaction had small effects on alpha and beta diversity but showed significant associations with groups of microorganisms such as *Acetobacterales* and *Saccharomycetes*, which are implicated in the disease sour rot and can contribute to wine characteristics within the fermentation process. This result will require further experimental validation in order to understand whether the associations with these microorganisms impact the susceptibility of clusters on an individual vine to sour rot and how the characteristics of the resulting wines are impacted. In addition, the role of the scion of grafted grapevines in shaping a vine’s microbial communities warrants investigation. An experimental design that makes use of reciprocal scion and rootstock combinations would allow for isolating the roles of the root and shoot systems in regulating the formation of a stable microbiome. 

## Figures and Tables

**Table 1:** Permutational Multivariate ANOVA results for bacterial and fungal communities using Unweighted UniFrac and Bray–Curtis dissimilarity metrics, respectively. Factors that are determined to be significant are bolded and interactions are denoted with the following abbreviations (R) rootstock, (C) compartment, and (I) irrigation.

	Bacterial Unweighted UniFrac			Fungal Bray–Curtis		
Factor	Pseudo-*F*	*p*-Value	R^2^	Pseudo-*F*	*p*-Value	R^2^
Rootstock (R)	*F*_3176_ = 1.124	0.252	0.011	***F*** **_3173_** ** = 2.178**	0.013	0.016
Compartment (C)	***F*** **_3176_** ** = 40.720**	**<0.001**	**0.407**	***F*** **_3173_** ** = 74.320**	**<0.001**	**0.550**
Irrigation (I)	*F*_2177_ = 1.107	0.273	0.007	*F*_2174_ = 1.203	0.247	0.006
Block	*F*_2177_ = 1.304	0.147	0.009	*F*_2174_ = 1.402	0.151	0.007
R × C	*F*_9170_ = 1.057	0.326	0.032	***F*** **_9167_** ** = 1.459**	**0.040**	**0.032**
R × I	*F*_6173_ = 1.058	0.324	0.021	*F*_6170_ = 1.146	0.261	0.017
C × I	*F*_6163_ = 1.009	0.417	0.020	*F*_6170_ = 0.936	0.526	0.014
C × R × I	*F*_18,161_ = 0.989	0.501	0.059	*F*_18,158_ = 0.998	0.473	0.044
Residual			0.433			0.313

**Table 2:** Three-way ANOVA model results for the abundance of *Acetobacterales* and *Saccharomycetes*. Factors that are determined to be significant are bolded and interactions are denoted with the following abbreviations (R) rootstock, (C) compartment, and (I) irrigation.

	*Acetobacterales*		*Saccharomycetes*	
Factor	F Value	*p*-Value	F Value	*p*-Value
Rootstock (R)	***F*_3128_ = 8.329**	**<0.001**	***F*_3124_ = 4.337**	**0.006**
Compartment (C)	*F*_3128_ = 0.002	1.000	*F*_3124_ = 0.003	1.000
Irrigation (I)	*F*_2129_ = 0.169	0.844	*F*_2125_ = 0.458	0.633
Block	*F*_2129_ = 0.347	0.708	*F*_2125_ = 0.311	0.733
R × C	***F*_9122_ = 2.111**	**0.033**	*F*_9118_ = 0.969	0.469
R × I	***F*_6125_ = 9.855**	**<0.001**	***F*_6121_ = 3.139**	**0.007**
C × I	*F*_6125_ = 0.046	1.000	*F*_6121_ = 0.114	0.995
C × R × I	***F*_18,113_ = 2.454**	**0.002**	*F*_18,109_ = 0.787	0.713

## Data Availability

All raw sequencing data is available on NCBI under BioProject ID PRJNA647904 and SRA accessions SRR12304683-SRR12304870. All code to reproduce the analysis and figures is available on GitHub at https://github.com/Kenizzer/Grapevine_Microbiota.

## Supplementary Materials

The following are available online at https://www.mdpi.com/2076-2607/9/1/92/s1, Figure S1. Taxonomic barplots for negative and positive controls, Figure S2. Rarefaction curves for bacterial and fungal samples, Figure S3. Alpha diversity metrics for bacterial and fungal samples, Figure S4. Venn diagrams show the overlap of ASVs between compartments for bacterial and fungal samples, Figure S5. Principal coordinates analysis showing the third axis based on bacterial unweighted Unifrac distance and fungal Bray–Curtis dissimilarity, Figure S6. Scatterplot of the abundance of *Acetobacterales* and *Saccharomycetes*, showing a strong positive correlation, Figure S7. Out of bag error estimate across values of the number of trees models attempting to predict rootstock, compartment, and rootstock by compartment jointly, Figure S8. Confusion matrix of model attempting to predict irrigation treatment, Figure S9. Absolute Log2 fold change values for ASVs associated with each source of variation, Table S1. Soil chemical analysis for the experimental vineyard, Table S2. Anova tables for bacterial alpha diversity metrics, Table S3. Anova tables for fungal alpha diversity metrics, Table S4. Model hyperparameters picked as optimal after tuning in Caret, Table S5. Output statistics for machine learning model predicting rootstock genotype, Table S6. Output statistics for machine learning model predicting compartment, Table S7. Output statistics for machine learning model jointly predicting rootstock and compartment.
