# Supplementary material for: Grapevine Microbiota Reflect Diversity among Compartments and Complex Interactions within and among Root and Shoot Systems
Source: Microorganisms. 2021 Jan 2;9(1):92. doi: 10.3390/microorganisms9010092 (PMC7823683; doi:10.3390/microorganisms9010092)
Supplement: Supplementary file 1 [file microorganisms-09-00092-s001.pdf]

Supplemental Materials

Figure S1. Taxonomic barplots for negative and positive controls used within the study. Above each bar is the number of reads after quality filtering for each of the samples. Mock DNA samples were ZymoBIOMICS™ Microbial community DNA standard (Cat No. D6305). Samples without a bar represent samples for which no reads passed quality filtering.

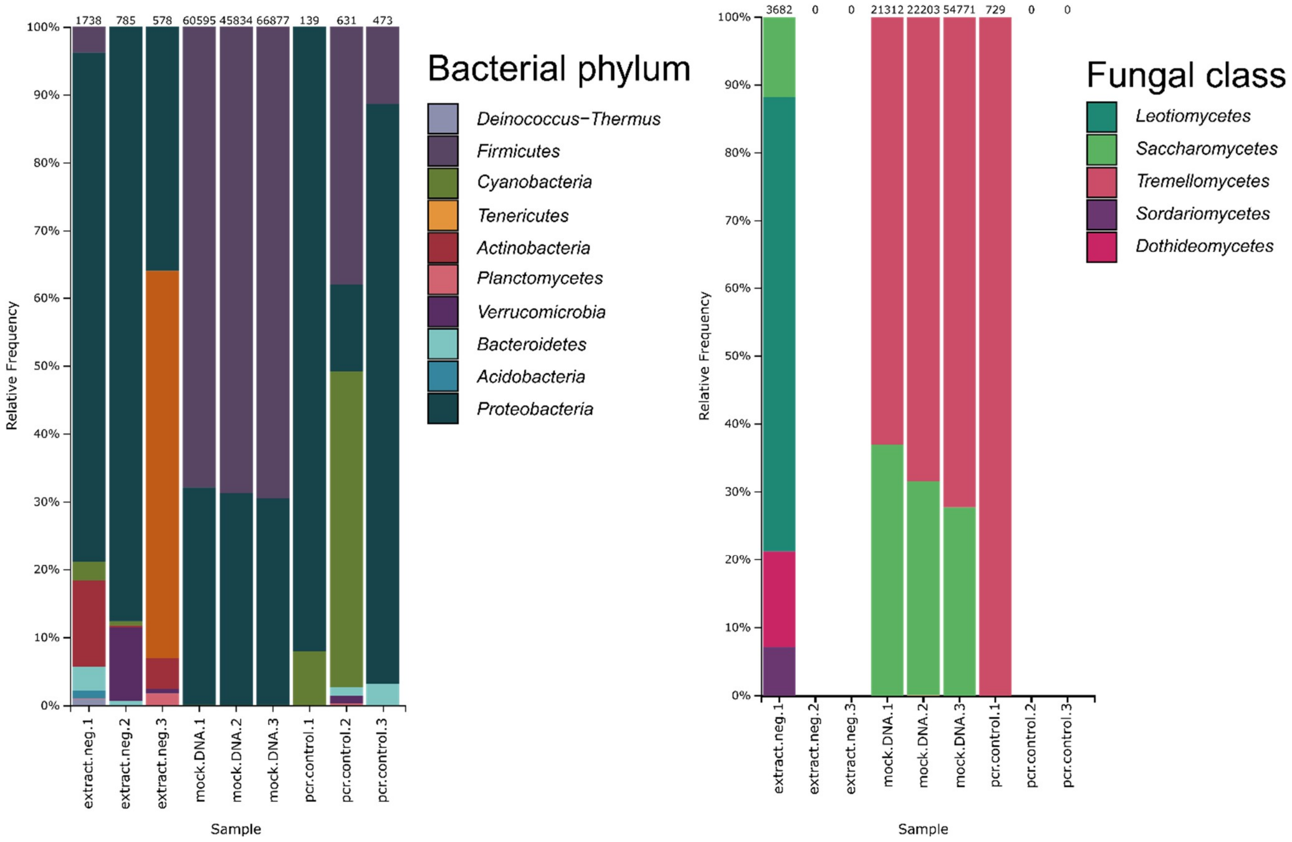

Figure S2. Rarefaction curves for A) bacterial and B) fungal samples.

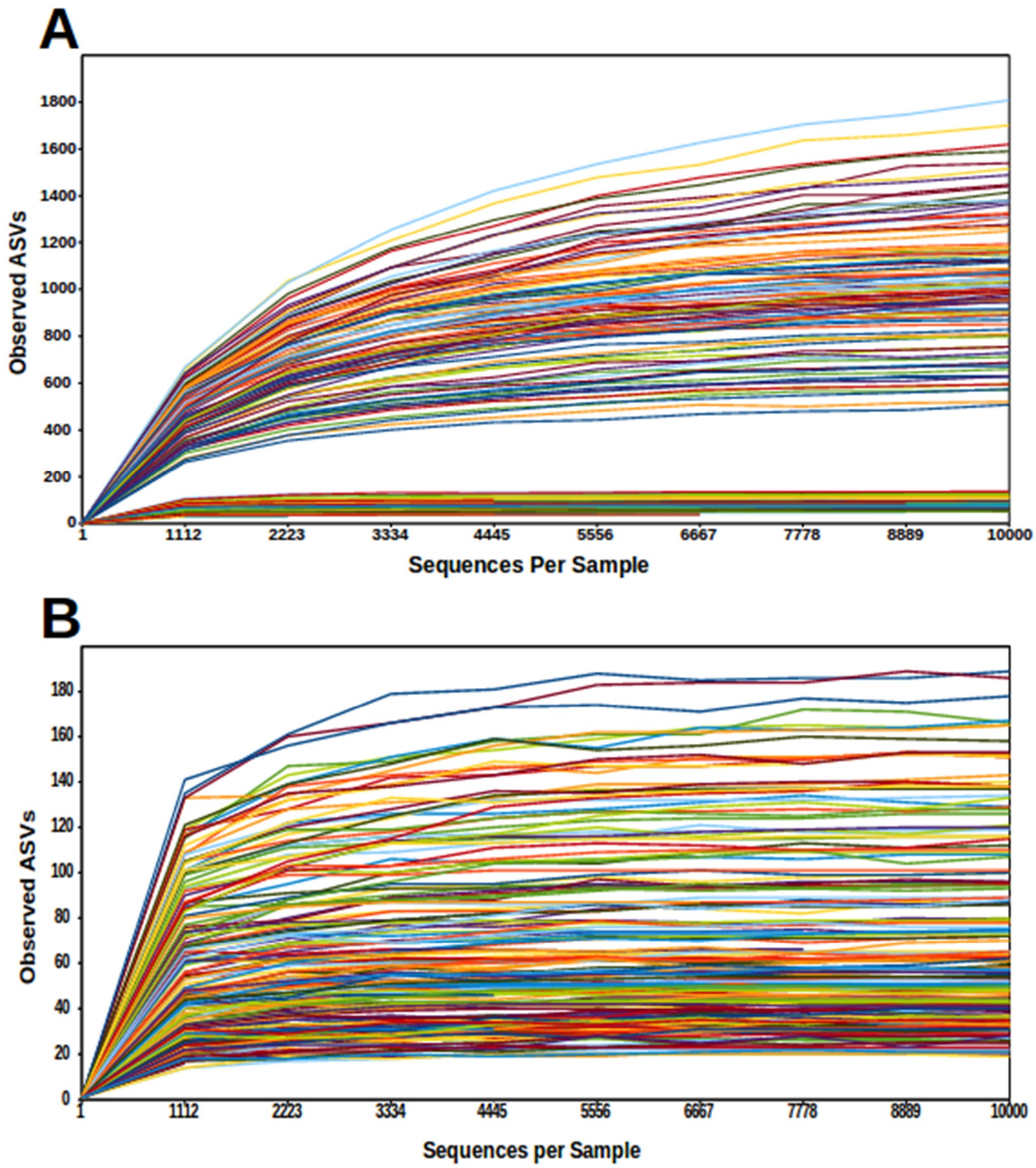

Figure S3. Alpha diversity metrics for A-C) bacterial and D-E) fungal communities by compartment.

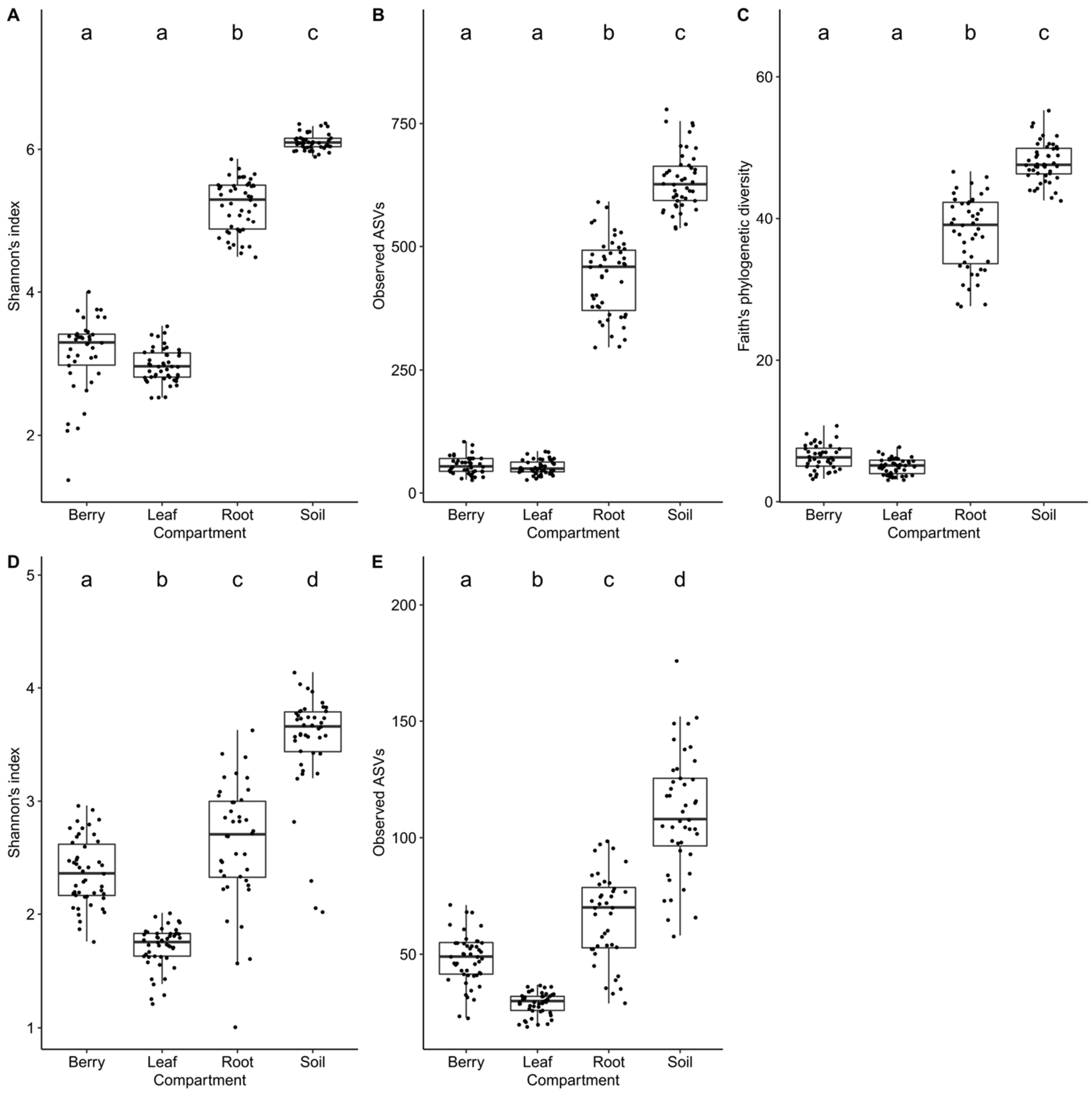

Figure S4. Venn diagrams depicting the overlap of ASVs between compartments and soil for A) bacterial and B) fungal samples.

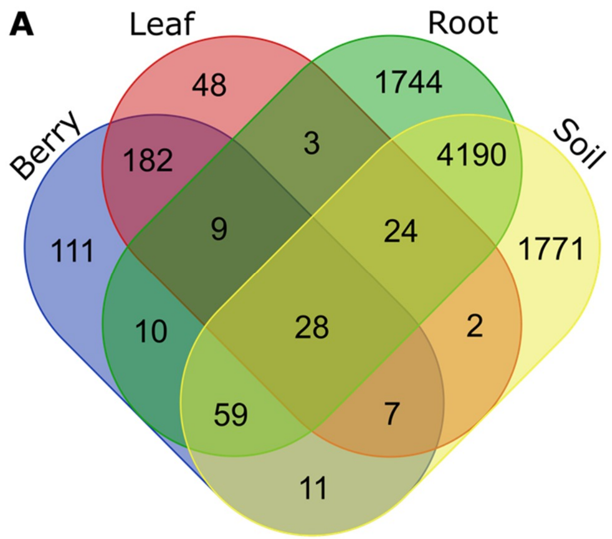

Figure S5. Principal coordinates analysis for A & B) Bacterial communities based on unweighted UniFrac distance and C & D) fungal communities based on Bray-Curtis dissimilarity.

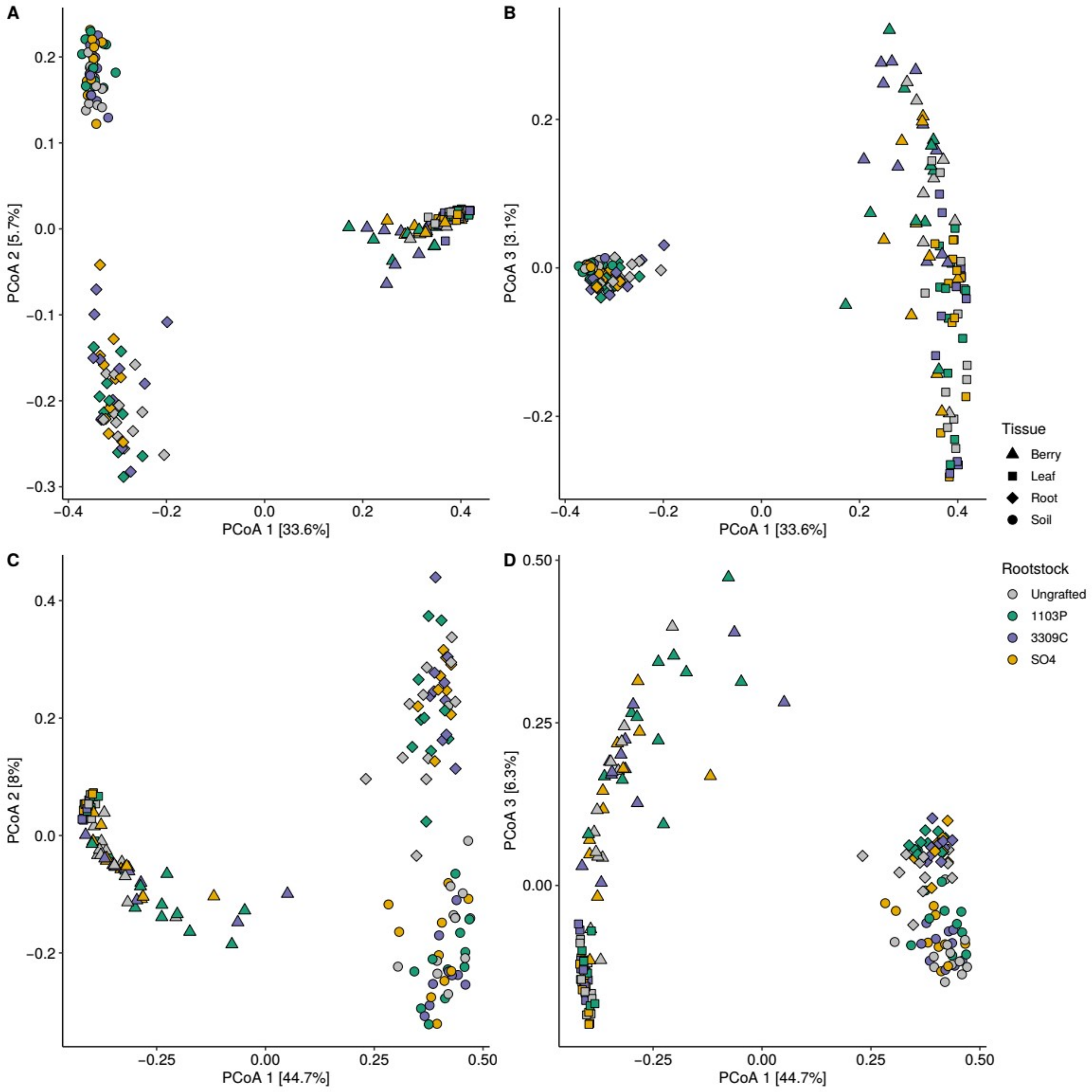

Figure S6. The abundance of *Acetobacterales* and *Saccharomycetes* show a strong positive correlation in the berry compartment. Spearman correlation coefficient and *P* value are reported for each compartment.

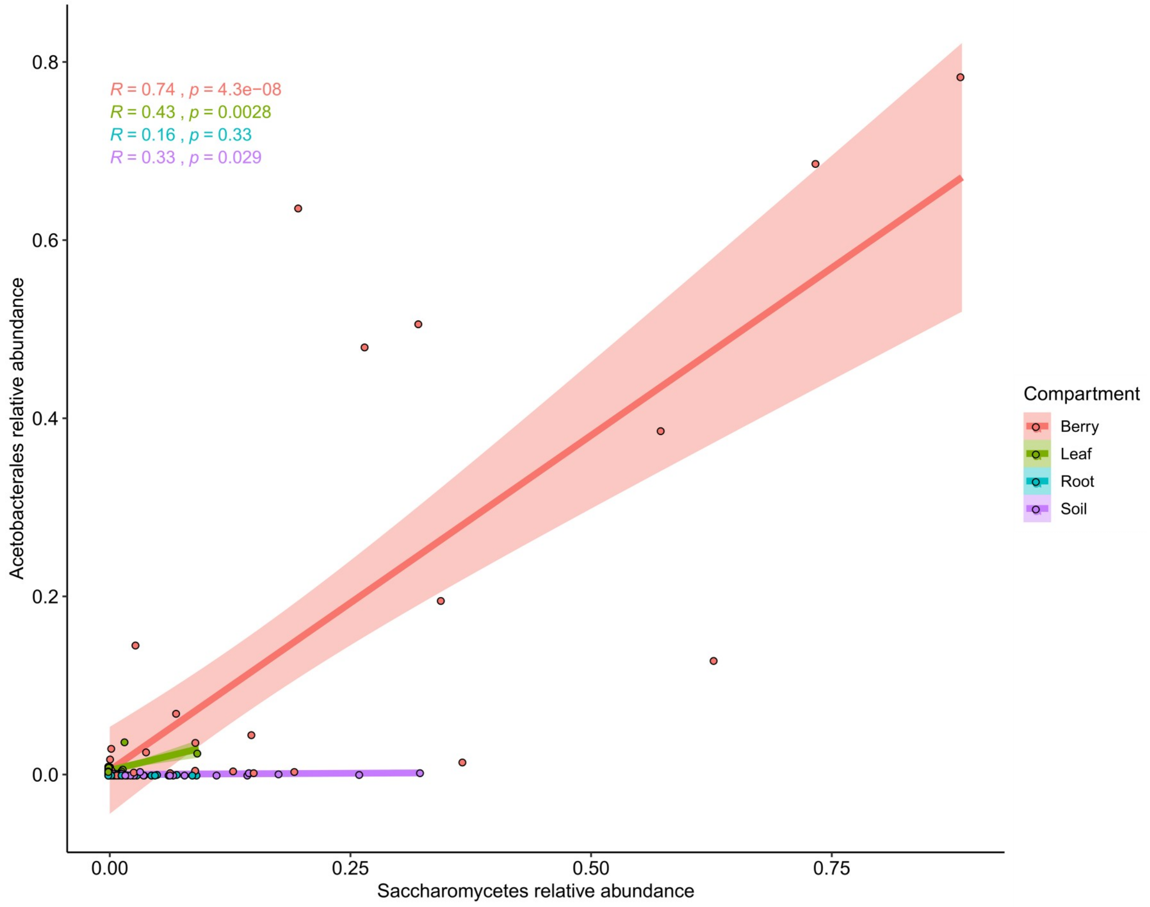

Figure S7. Out of Bag error estimate for different values of the number of trees across models attempting to predict rootstock, compartment, and rootstock by compartment jointly.

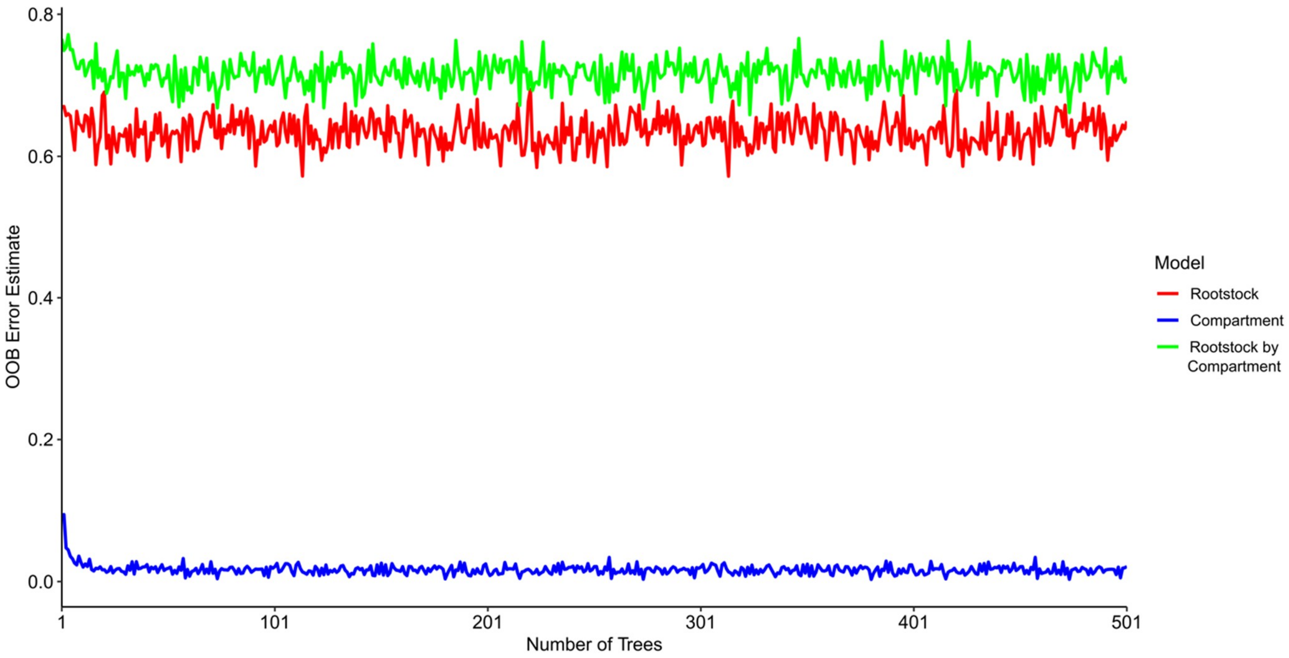

Figure S8. Machine learning analysis attempting to predict the irrigation treatment a given sample was under. Irrigation treatments were full replacement of evapotranspiration, intermediate replacement (50%), and nonirrigated.

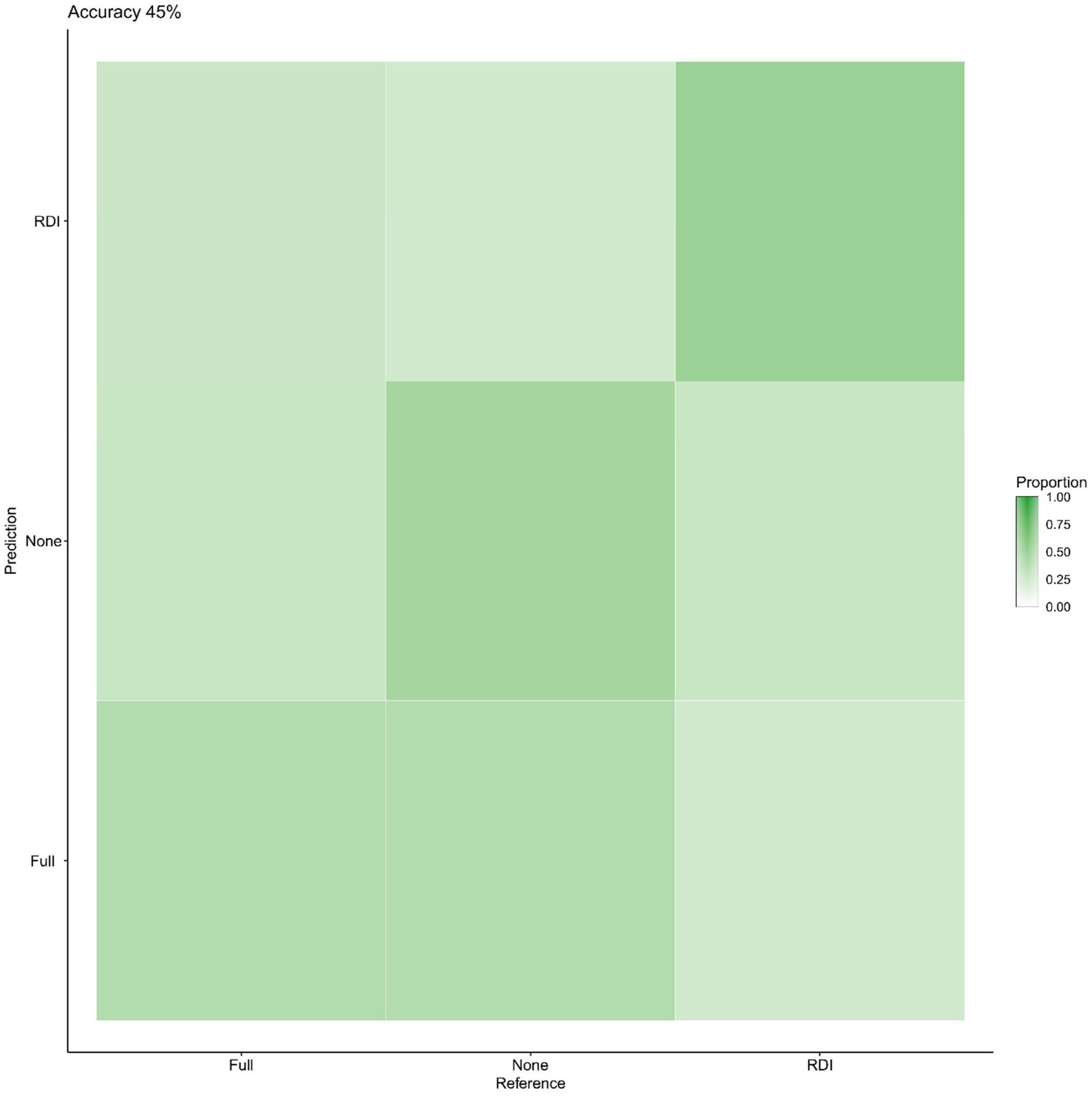

Figure S9. Violin plot of extracted absolute Log_2 fold change values for ASVs associated with each source of variation.

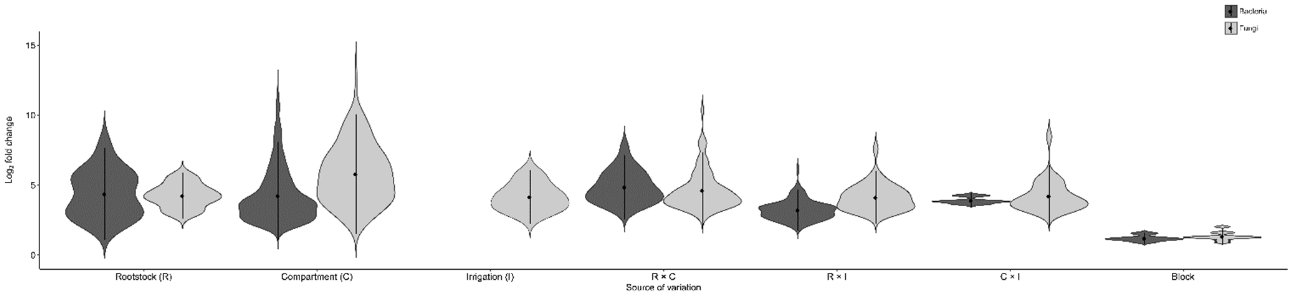

Table S1. Soil analysis of samples collected from the University of Missouri Southwest Research Center in Mount Vernon, MO. Analysis was completed at the University of Arkansas Fayetteville Agricultural Diagnostic Laboratory according to the established protocols (Sikora and Kissel 2014; Zhang et al. 2014). Values are reported in mg/kg (ppm) with the exception of pH which is reported in the standard scale.

Sample	pH	P	K	Ca	Mg	S	Na	Fe	Mn	Zn	Cu	B
**Block 3N**	7.2	123	197	2590	311	12	7	82	83	60	3.4	1.5
**Block 3I**	7.4	128	130	2873	296	11	14	83	76	30	4.4	1.2
**Block 3F**	7.0	138	131	2633	287	13	12	99	65	36	5.5	1.5
**Block 2I**	7.1	112	124	2793	297	14	15	82	99	49	33.2	1.7
**Block 2F**	7.2	95	100	2502	321	11	15	93	62	35	7.5	1.4
**Block 2R**	7.2	189	191	2849	314	14	7	104	76	34	15.1	1.6
**Block 1I**	7.5	178	127	3302	311	12	16	90	83	50	11.2	1.8
**Block 1N**	7.1	178	160	2798	262	12	7	110	69	27	10.3	1.5
**Block 1F**	7.3	186	154	2857	348	14	14	104	81	30	12.3	1.6

Table S2. Anova tables for bacterial alpha diversity metrics.

**Faith's phylogenetic diversity**				**Inverse Simpson's index**		
Factor	SS	*F* value	*p*-value	SS	*F* value	*p*-value
Intercept	**96560.477**	$\mathbf{F_{1,180} = 9936.466}$	**< 0.001**	**1493749.18**	$\mathbf{F_{1,180} = 1607.342}$	**< 0.001**
Compartment (C)	**64538.239**	$\mathbf{F_{3,178} = 2213.749}$	**< 0.001**	**2357695.10**	$\mathbf{F_{3,178} = 845.662}$	**< 0.001**
Rootstock (R)	32.471	$F_{3,178} = 1.114$	0.346	3528.06	$F_{3,178} = 1.265$	0.289
Irrigation (I)	47.609	$F_{2,179} = 2.450$	0.090	1625.56	$F_{2,179} = 0.875$	0.419
Block	**69.621**	$\mathbf{F_{2,179} = 3.582}$	**0.031**	**6220.15**	$\mathbf{F_{2,179} = 3.347}$	**0.038**
C X R	72.984	$F_{9,172} = 0.834$	0.586	4075.24	$F_{9,172} = 0.487$	0.881
C X I	122.987	$F_{6,175} = 2.109$	0.056	4011.82	$F_{6,175} = 0.719$	0.635
R X I	73.418	$F_{6,175} = 1.259$	0.281	3806.05	$F_{6,175} = 0.683$	0.664
C X R X I	89.634	$F_{18,163} = 0.5124$	0.948	14491.03	$F_{18,163} = 0.866$	0.620
Residuals	1273.030			121742.09		

**Observed ASVs**				**Shannon's Diversity**		
Factor	SS	*F* value	*p*-value	SS	*F* value	*p*-value
Intercept	**14178127.06**	$\mathbf{F_{1,180} = 5311.543}$	**< 0.001**	**3109.42**	$\mathbf{F_{1,180} = 24224.460}$	**< 0.001**
Compartment (C)	**11212803.39**	$\mathbf{F_{3,178} = 1400.215}$	**< 0.001**	**321.84**	$\mathbf{F_{3,178} = 835.780}$	**< 0.001**
Rootstock (R)	6316.28	$F_{3,178} = 0.789$	0.502	0.08	$F_{3,178} = 0.195$	0.899
Irrigation (I)	7320.21	$F_{2,179} = 1.371$	0.257	0.04	$F_{2,179} = 0.148$	0.862
Block	13075.72	$F_{2,179} = 2.449$	0.090	0.28	$F_{2,179} = 1.075$	0.344
C X R	12698.48	$F_{9,172} = 0.529$	0.852	0.24	$F_{9,172} = 0.206$	0.993
C X I	17678.90	$F_{6,175} = 1.104$	0.364	0.64	$F_{6,175} = 0.833$	0.546
R X I	22167.44	$F_{6,175} = 1.384$	0.226	1.04	$F_{6,175} = 1.355$	0.238
C X R X I	31377.77	$F_{18,163} = 0.653$	0.851	1.52	$F_{18,163} = 0.658$	0.846
Residuals	349678.95			16.81		

Table S3. Anova tables for fungal alpha diversity metrics.

Inverse Simpson's index				Observed ASVs			Shannon's Diversity		
Factor	SS	*F* value	*p*-value	SS	*F* value	*p*-value	SS	*F* value	*p*-value
Intercept	**13074.903**	**$F_{1,176}$ = 602.832**	**<0.001**	**625159.756**	**$F_{1,176}$ = 2236.156**	**<0.001**	**1010.141**	**$F_{1,176}$ = 6451.465**	**<0.001**
Compartment (C)	**6559.548**	**$F_{3,174}$ = 100.812**	**<0.001**	**161851.084**	**$F_{3,174}$ = 192.977**	**<0.001**	**74.877**	**$F_{3,174}$ = 159.407**	**0.001**
Rootstock (R)	**219.702**	**$F_{3,174}$ = 3.377**	**0.020**	1454.586	$F_{3,174}$ = 1.734	0.163	0.527	$F_{3,174}$ = 1.122	0.343
Irrigation (I)	87.568	$F_{2,175}$ = 2.019	0.137	1288.001	$F_{2,175}$ = 2.304	0.104	0.661	$F_{2,175}$ = 2.112	0.125
Block	115.236	$F_{2,175}$ = 2.657	0.074	555.667	$F_{2,175}$ = 0.994	0.373	0.697	$F_{2,175}$ = 2.224	0.112
C X R	**427.601**	**$F_{9,168}$ = 2.191**	**0.027**	3466.575	$F_{9,168}$ = 1.378	0.205	0.846	$F_{9,168}$ = 0.600	0.795
C X I	96.701	$F_{6,171}$ = 0.743	0.616	2182.995	$F_{6,171}$ = 1.301	0.261	1.129	$F_{6,171}$ = 1.202	0.310
R X I	38.709	$F_{6,171}$ = 0.297	0.937	1668.859	$F_{6,171}$ = 0.995	0.432	0.625	$F_{6,171}$ = 0.666	0.678
C X R X I	212.811	$F_{18,159}$ = 0.545	0.931	4385.173	$F_{18,159}$ = 0.871	0.613	1.740	$F_{18,159}$ = 0.617	0.881
Residuals	2754.520			35505.249			19.885		

Table S4. Selected model hyperparameters showing optimal results in Caret.

Model	mtry	Minimum node size	Number of trees
**Rootstock**	2827	5	314
**Compartment**	949	5	381
**Rootstock × Compartment**	7522	10	324

Table S5. Output statistics for machine learning model predicting rootstock genotype.

Prediction: Rootstock				
**Class**	**Ungrafted**	**1103P**	**3309C**	**SO4**
**Precision**	0.583	0.500	0.333	0.273
**Recall**	0.583	0.364	0.333	0.375
**F1**	0.583	0.421	0.333	0.316

Table S6. Output statistics for machine learning model predicting compartment.

Prediction: Compartment				
**Class**	**Berry**	**Leaf**	**Root**	**Soil**
**Precision**	1	1	1	1
**Recall**	1	1	1	1
**F1**	1	1	1	1

Table S7. Output statistics for machine learning model jointly predicting rootstock and compartment.

Prediction: Rootstock by Compartment

Class	Berry 1103P	Berry 3309C	Berry SO4	Berry Ungrafted	Leaf 1103P	Leaf 3309C	Leaf SO4	Leaf Ungrafted	Root 1103P	Root 3309C	Root SO4	Root Ungrafted	Soil 1103P	Soil 3309C	Soil SO4	Soil Ungrafte d
**Precis ion**	0.333	NA	0	0	0.5	0.200	0	0	1	1	1	1	0.500	0.333	NA	0.500
**Recall**	0.667	0	0	0	0.667	0.333	0	0	1	1	1	1	0.333	1	0	0.667
**F1**	0.444	NA	NA	NA	0.571	0.250	NA	NA	1	1	1	1	0.400	0.500	NA	0.571

Citations

Sikora, F.J., and D.E. Kissel. 2014. Soil pH. In: F.J. Sikora and K.P. Moore, editors, Soil test methods from the southeastern United States. Southern Coop. Ser. Bull. 419. p. 48–53.

Zhang, H., Hardy, D.H., Mylavarapu, R., and Wang, J.J. 2014. Mehlich-3. In: F.J. Sikora and K.P. Moore, editors, Soil test methods from the southeastern United States. Southern Coop. Ser. Bull. 419. p. 101–110.
